# In Vitro and In Silico Evaluation of the Anti-Aging Potential of *Eugenia uniflora* UAE Extracts

**DOI:** 10.3390/molecules30153168

**Published:** 2025-07-29

**Authors:** Desy Muliana Wenas, Berna Elya, Sutriyo Sutriyo, Heri Setiawan, Rozana Othman, Syamsu Nur, Nita Triadisti, Fenny Yunita, Erwi Putri Setyaningsih

**Affiliations:** 1 Faculty of Pharmacy, National Institute of Science and Technology, Jakarta 12630, Indonesia; desywenas@istn.ac.id (D.M.W.); erwi@istn.ac.id (E.P.S.); 2 Faculty of Pharmacy, Universitas Indonesia, Depok 16424, Indonesia; sutriyo@farmasi.ui.ac.id (S.S.); heri.setiawan@farmasi.ui.ac.id (H.S.); 3 Department of Pharmaceutical Chemistry, Faculty of Pharmacy, Universiti Malaya, Kuala Lumpur 50603, Malaysia; rozanaothman@um.edu.my; 4 Department of Pharmaceutical Chemistry, Faculty of Pharmacy, Universitas Almarisah Madani, Makassar 90245, Indonesia; syamsunur19@gmail.com; 5 Department of Biology Pharmacy, Faculty of Pharmacy, Universitas Muhammadiyah Banjarmasin, Banjarmasin 70115, Indonesia; triadisti@umbjm.ac.id; 6 Department of Pharmacology, Faculty of Medicine, Tarumanagara University, Jakarta 11440, Indonesia; fenny@fk.untar.ac.id

**Keywords:** Anti-elastase, antioxidant, dewandaru, in silico, LC MS, molecular docking

## Abstract

Skin aging is a natural biological process that can be accelerated by free radical induction, leading to a reduction in skin elasticity and the formation of wrinkles due to the depletion of elastin. *Eugenia uniflora* (dewandaru) is a promising plant believed to possess anti-aging properties, primarily attributed to its major constituents, myricitrin and quercetin. This study aimed to investigate the anti-elastase and antioxidant properties of *Eugenia uniflora* stem bark, ripe fruit, and seed extracts. Extracts were obtained using an ultrasound-assisted extraction (UAE) method with 70% ethanol. Quantitative phytochemical analysis involved measuring the total phenolic content (TPC), total flavonoid content (TFC), and antioxidant activity. Bioactive constituents were identified using LC-MS analysis, and their interactions with target enzymes were further evaluated through in silico molecular docking. The results demonstrated that the *E. uniflora* seed extract exhibited the highest antioxidant activity, with an IC_50_ of 5.23 µg/mL (DPPH assay) and a FRAP value of 3233.32 µmol FeSO_4_/g. Furthermore, the ethanolic seed extract showed significant anti-elastase activity with an IC_50_ of 114.14 µg/mL. Molecular docking predicted strong potential for several compounds as pancreatic elastase inhibitors, including 5-phenylvaleric acid, 2-(3-phenylpropyl)phenol, n-amylbenzene, 2-aminoadipic acid, and traumatin, each showing a prediction activity (PA) value exceeding 0.6. Notably, these compounds also exhibited inhibitory activity against tyrosinase. These findings collectively underscore the significant promise of *E. uniflora* seed extract as a novel and natural candidate for pharmacocosmeceutical product development, particularly for anti-aging applications.

## 1. Introduction

As a tropical country, Indonesia experiences high exposure to ultraviolet (UV) light, which can lead to skin damage, including wrinkles and loss of elasticity [1]. UV radiation promotes the formation of free radicals, and at elevated concentrations, these can damage key skin proteins like elastin and collagen. Specifically, UVA radiation has been shown to notably upregulate the gene expression of matrix metalloproteinase-1 (MMP-1) and elastase in skin fibroblasts [2]. Elastases are serine proteolytic enzymes that degrade various connective tissue proteins in the skin, such as elastin. Skin fibroblast elastase, in particular, is primarily responsible for breaking down elastic fibers, thereby triggering wrinkle formation [3]. Consequently, inhibiting elastase activity represents a viable strategy to protect against skin aging [1]. Furthermore, the accumulation of reactive oxygen species (ROS) in skin cells can lead to damage, increased pigmentation, accelerated skin aging, and oxidative stress. Plant-derived antioxidants offer a potential approach to counteract the skin aging and hyperpigmentation induced by ROS [3]. *Eugenia uniflora* (dewandaru) is known in Indonesia for its high content of phenolics and flavonoids, making it a promising source of potential anti-aging agents.

Dewandaru originates from Brazil and South America and is widely distributed in Southeast Asia, including Indonesia. The leaves of this plant have been utilized in traditional medicine to address conditions such as hypertension, digestive disorders, inflammation, cough, fever, and sore throat. The biological activities have been investigated, such as antidiarrheal, antifungal, and anti-inflammatory properties [1]. The plant of *E. uniflora* has abundant ethnopharmacological properties. The essential oil of *E. uniflora* is utilized for industrial cosmetics due to its unique scent with citric and exotic green notes, making it a valuable perfumery ingredient [2]. The fruit has a shape like a small pumpkin, which can be eaten fresh or used in liquors, jams, jellies, ice creams, and juices [3]. Bark extracts contain flavonoids, tannins, coumarins, terpenes, and sterols and exhibit analgesic activity [1]. The ripe fruit of *E. uniflora* is proven to contain high concentrations of anthocyanins and carotenoids, while its extract has DPPH antioxidant activity [4]. The fruit pulp of *E. uniflora* is known to contain quercetin and myricetin glycosides, while the pericarp contains heptenone, linalool, maaliol, and curzerene [5]. The seeds of *E. uniflora* have phenolic compounds five times higher than their pericarp fruit [6]. However, the fruits of *E. uniflora*, along with their seeds and bark, have not been explored in Indonesia. The seed also accumulates significant secondary metabolites such as flavonoids [7].

Prior reports have documented studies on the anti-elastase, anti-tyrosinase, and antioxidant actions of species belonging to the *Eugenia* genus. *Eugenia dysenterica* ethanolic leaf extract is known for its elastase and tyrosinase inhibition activity [8]. Similarly, an ethanolic leaf extract of *Eugenia punicifolia* demonstrated a notable capacity to inhibit human neutrophil elastase activity, an enzyme particularly relevant to inflammatory processes and tissue degradation [9]. *Eugenia leitonii, Eugenia involucrata, Eugenia brasiliensis,* and *Eugenia myrcianthes* leaf extracts were able to exhibit an antioxidant effect that was capable of neutralizing radicals such as peroxyl, superoxide, and hypochlorous acid [10]. Given that *E. uniflora* shares the same genus with *E. dysenterica* and *E. punicifolia*, it is biologically plausible that *E. uniflora* also possesses the phytochemical composition and structural features necessary to exert similar elastase inhibitory effects. The presence of shared secondary metabolites (e.g., flavonoids, phenolics, terpenes) across closely related species often leads to similar pharmacological properties. Nonetheless, its ability to inhibit the elastase activity of *E. uniflora*, especially from Indonesia, has not been observed.

Ultrasound-assisted extraction (UAE) is a modern green extraction technique that enhances the release of plant secondary metabolites. UAE uses ultrasonic waves to create a small bubble cavitation in a solvent. The interaction of mechanical forces and their thermal influence are responsible for its extraction ability. This causes the cell wall to be disrupted, which lowers particle size, allows the solvent to have greater penetration into the plant cell, and increases mass transfer through the cell membrane [11]. Compared to conventional methods, UAE offers shorter extraction times, improved yield, and better preservation of thermolabile compounds [12]. Furthermore, the integration of in silico tools such as molecular docking has accelerated the identification of bioactive compounds by predicting their interaction with target enzymes such as elastase and tyrosinase. Recent advances in computational biology, particularly molecular docking, have accelerated the identification of potential bioactive compounds by predicting their binding affinity and interactions with target enzymes [13].

Therefore, it is essential to investigate the phytochemical composition and bioactivities of *Eugenia uniflora* UAE extracts derived from the bark, fruit, and seeds, particularly their antioxidant and elastase-inhibitory potential. Among these, the extract exhibiting the most potent bioactivity will undergo further evaluation through an elastase inhibition assay to confirm its anti-aging potential. The bark, fruits, and seeds are considered promising sources of natural anti-aging agents. The most active extract will then be subjected to phytochemical profiling using liquid chromatography–mass spectrometry (LC-MS) to identify its major constituents. Subsequently, the identified compounds will be evaluated through in silico molecular docking to predict their binding affinity and potential inhibitory mechanisms against key aging-related enzymes, specifically elastase and tyrosinase. This integrative approach aims to reveal lead compounds with dual activity, supporting the development of natural source cosmeceuticals targeting oxidative stress, elastin degradation, and melanin overproduction that may serve as candidates for the development of cosmeceutical or therapeutic agents targeting skin aging and pigmentation disorders.

## 2. Results

### 2.1. Extraction and Phytochemical Screening

Ten grams of bark, ripe fruit, and seeds of *Eugenia uniflora* were extracted using ultrasound-assisted extraction (UAE). The UAE yielded 12.1% bark extract, 16.6% ripe fruit extract, and 16.1% ripe fruit seed extract, respectively. Qualitative phytochemical screening of bark, fruit, and seed dewandaru UAE extracts showed the presence of various secondary metabolite compounds such as saponins, tannins, terpenoids, alkaloids, and flavonoids (Table 1).

The total phenolic content of the *Eugenia uniflora* bark, fruit, and seed extracts was held with the Folin–Ciocalteu reagent. The flavonoid was determined using the aluminum chloride colorimetric method. The TPC and TFC of seed extracts were higher than those of bark and fruit UAE extracts (Table 2).

### 2.2. Antioxidant Activities

The relationship between inhibition percentage and the concentration of extracts is presented in linear regression equations. The highest DPPH radical scavenging ability among *Eugenia uniflora* extracts was the seed extract, which still had lower antioxidant activity than quercetin as a positive control. The amount of IC_50_ in the extract was slightly higher than the positive control, which means the seed extract has fewer antioxidants compared to quercetin. The FRAP test results were calculated from the equation of the calibration curve of iron (II) sulfate heptahydrate. The seed extract had a higher FRAP value than other extracts, even greater than the quercetin positive control (Table 3). A higher FRAP value indicates greater potential antioxidant activity.

### 2.3. Anti-Elastase Activity

Among the three extracts, the seed extract was chosen to be tested for its elastase inhibition activity. Kinetic measurements of porcine pancreatic elastase (PPE) activity were determined using Suc-(Ala)3-p-nitroanilide as a substrate and polyphenolic compounds as inhibitors. The experimental result showed that the *E. uniflora* ripe seed UAE extract exhibited elastase inhibition activity with an IC_50_ value of 114.08 ± 0.73 µg/mL (Table 4), and the IC_50_ of quercetin (positive control) was 45.39 ± 0.81 µg/mL. The percentage inhibition data are expressed as the mean and standard deviation (SD).

### 2.4. LC-MS

The LC-MS result shows that the seed extract contained 27 compounds (Table 5). The dominant compounds found in *E. uniflora* seed extract are citric acid, two aminoadipic acids, abscisic acid, one stearoylglycerol, quinic acid, gallic acid, oleamide, myricitrin, and myricetin.

### 2.5. Screening Phytocompounds for Antiaging Bioactivity

Among the 141 chemical compounds identified, 83 are present in the PubChem NCBI database and 26 were found to possess pancreatic elastase inhibitory properties. The compounds that had a value lower than 0.6 are 5-phenylvaleric acid, valine, L-phenylalanine, 2-(3-phenylpropyl)phenol, n-amylbenzene, 2-aminoadipic acid, and traumatin.

### 2.6. Target Compound Pharmacokinetics Characteristics

The prediction of the pharmacokinetic characteristics (absorption, distribution, metabolism, excretion, and toxicity) of the target compounds was evaluated and is presented in Table 6. The Caco-2 cell line, derived from human epithelial colorectal adenocarcinoma, is widely used as an in vitro model for the intestinal epithelium to predict oral drug absorption due to its morphological and functional similarities to human enterocytes [14]. The compounds studied exhibited Caco-2 permeability ranging from −4.2 to −5.91, indicating low intestinal permeability.

Oral bioavailability predictions demonstrated that traumatin (20% predicted bioavailability) is likely non-bioavailable, while 5-phenylvaleric acid, n-amylbenzene, and 2-(3-phenylpropyl)phenol exhibited 50% predicted bioavailability, suggesting moderate systemic exposure via oral administration [15].

All compounds were predicted to be non-inhibitors and non-substrates of P-glycoprotein (P-gp), an ATP-binding cassette (ABC) efflux transporter. P-gp plays a critical role in drug efflux across biological barriers such as the intestinal epithelium and the blood–brain barrier, affecting bioavailability and drug–drug interactions [16]. The modulation of P-gp activity has substantial implications in drug absorption, distribution, and resistance [4]. In vitro and transgenic knockout mouse models are commonly used for evaluating P-gp substrate and inhibitor properties [17].

Skin permeability, expressed as log Kp (cm/h), is a critical parameter in assessing both the efficacy of topically applied products and the systemic exposure associated with dermal absorption. A log Kp > −2.5 is considered indicative of low skin permeability [18]. L-phenylalanine and traumatin exhibited low predicted permeability, while the remaining compounds demonstrated relatively higher transdermal penetration potential.

All compounds were predicted to penetrate the blood–brain barrier (BBB). The BBB restricts the entry of exogenous compounds into the central nervous system (CNS), and its permeability is essential for evaluating CNS drug efficacy and neurotoxicity [19]. BBB permeability was assessed using the log permeability–surface area product (log PS), where compounds are classified as CNS^+^ (log PS ≥ −2) or CNS^−^ (log PS ≤ −3) [20]. However, efflux via P-gp and other transporters may reduce net brain exposure regardless of intrinsic permeability.

The fraction unbound (FU) in plasma reflects the proportion of a drug that is not bound to plasma proteins and is pharmacologically active. Only the unbound fraction can cross cellular membranes, undergo metabolism, or exert therapeutic effects [21].

The volume of distribution at steady state (VDss) indicates the theoretical volume required to contain the drug at the same concentration as in plasma. A high VDss suggests significant tissue distribution, while a low VDss implies confinement to the vascular compartment. Compounds with log VDss < −0.15 (VDss < 0.71 L/kg) are classified as low, and those with log VDss > 0.45 (VDss > 2.81 L/kg) as high [22]. Distribution can be altered by physiological factors such as renal failure and dehydration. Breast cancer resistance protein (BCRP), an ABC efflux transporter similar to P-gp, was also considered. BCRP limits drug accumulation in tissues such as the brain, placenta, and testes and is an important determinant in multidrug resistance and oral bioavailability [23].

Cytochrome P450 (CYP) enzymes are key to the hepatic metabolism of drugs. The major isoforms (CYP1A2, CYP2C19, CYP2C9, CYP2D6, and CYP3A4) account for the metabolism of over 90% of clinically used drugs [24]. Compounds were assessed for potential inhibition and substrate activity toward these isoforms. A compound is considered an inhibitor if it reduces enzyme activity by 50% at <10 µM concentration [25]. Inhibiting or inducing these enzymes may result in altered drug exposure and potential toxicity.

Organic anion-transporting polypeptides (OATP1B1 and OATP1B3) are hepatic uptake transporters that facilitate the entry of xenobiotics and drugs into hepatocytes, impacting their metabolism and clearance [26]. The total clearance (CLtot), expressed in mL/min/kg, is a primary pharmacokinetic parameter representing the volume of plasma from which the drug is completely removed per unit time. It is influenced by both hepatic metabolism and renal excretion and directly impacts dosing regimens and therapeutic window [27].

The elimination half-life (t_1_/_2_) refers to the time needed for the plasma concentration of a drug to reduce by 50% and is crucial for determining dosing intervals. Compounds with t_1_/_2_ ≥ 3 h are considered to have extended systemic retention (Class 1), whereas those with t_1_/_2_ < 3 h are rapidly eliminated (Class 0) [28].

Finally, the compounds were evaluated for the inhibition of organic cation transporter 2 (OCT2), located on the basolateral membrane of renal proximal tubules. OCT2 is involved in the active renal secretion of cationic drugs. The inhibition of OCT2 can affect renal drug clearance and lead to the accumulation of co-administered drugs [29].

### 2.7. Toxicity Assessment

According to Table 7, the in silico toxicity assessment of the target compounds—5-phenylvaleric acid, valine, L-phenylalanine, n-amylbenzene, 2-aminoadipic acid, traumatin, and 2-(3-phenylpropyl)phenol—revealed favorable safety profiles. All compounds were predicted to be non-mutagenic (AMES test) and non-toxic to avian species. With the exception of valine, which exhibited potential bee toxicity, the remaining compounds were considered safe for pollinators. Bioconcentration factor (BCF) values indicated low to moderate bioaccumulation potential, with n-amylbenzene showing the highest BCF value (2.68), suggesting a need for further environmental consideration. Compared to retinoic acid as the reference compound, the overall toxicity profiles of the target compounds support their candidacy for further development in pharmaceutical applications, particularly in formulations with anti-aging potential.

### 2.8. Analysis of Docking Elastase

**Figure 1:**
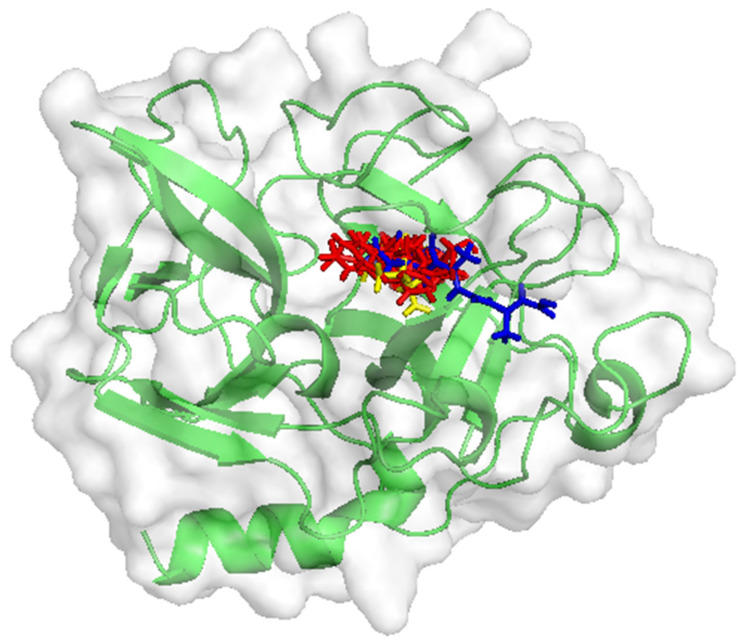
Interaction target compound and elastase. The elastase protein structure (green), the target compound structure (red), native ligand as inhibitor (blue), and retinoic acid (yellow).

Based on the results of the docking analysis, all of the compounds bind in the elastase inhibitor region, specifically at the SER195 and Val216 residues. In addition, several inhibitor residues were also found to be active site residues of the target. Both 5-phenylvaleric acid and 2-(3-phenylpropyl)phenol bound to the HIS57 residue, which is also an active site residue for retinoic acid and elastase inhibitors. The Ser214 residue is the binding site for L-phenylalanine, which is identified as a residue in the retinoic acid and elastase inhibitor binding region (Figure 1, Table 8).

**Figure 2:**
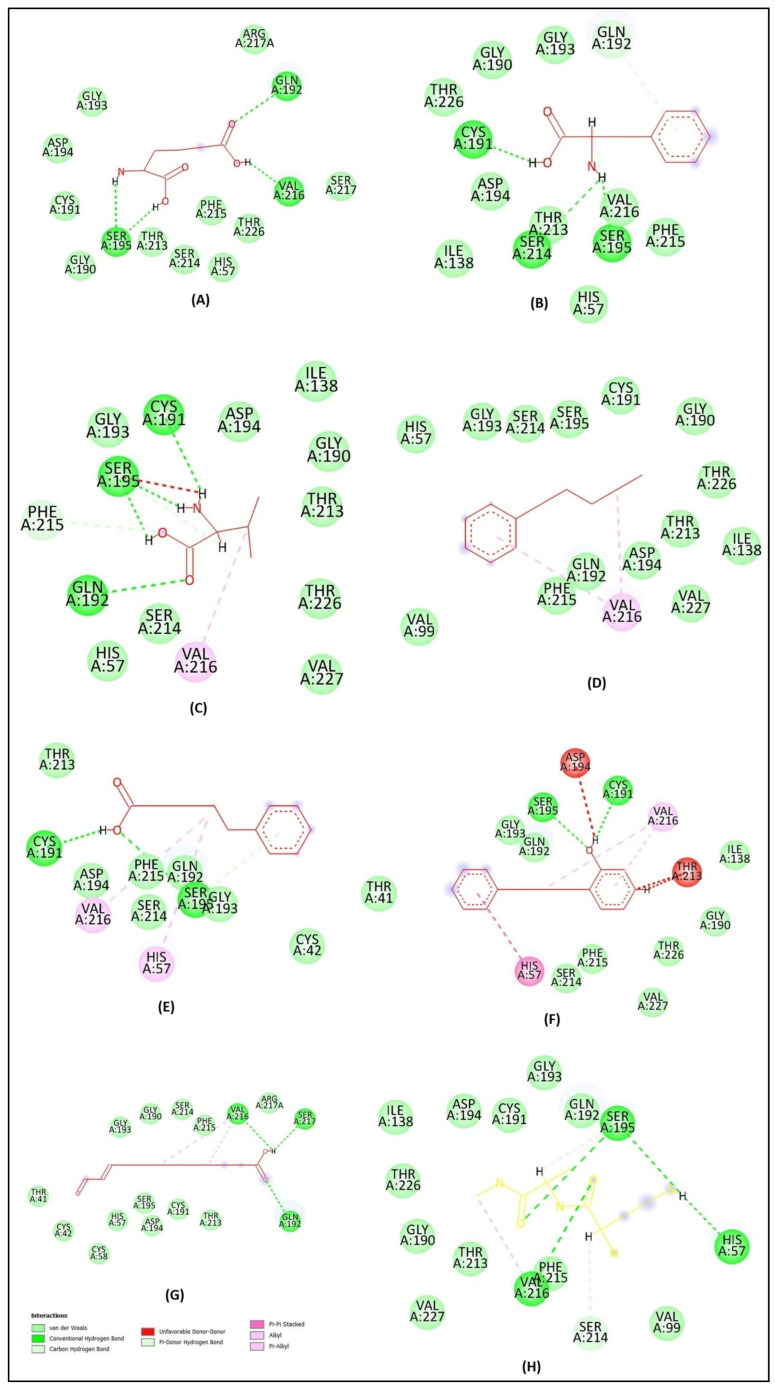
Two-dimensional diagram of the interaction between 2-aminoadipic acid (**A**), L-phenylalanine (**B**), valine (**C**), n-amylbenzene (**D**), 5-phenylvaleric acid (**E**), 2-(3-phenylpropyl)phenol (**F**), traumatin (**G**), and retinoic acid (**H**) with the elastase.

The molecular docking analysis of 2-aminoadipic acid with elastase revealed four hydrogen bonds involving the key residues Ser195, Val216, and Gln192. Interestingly, although Val216 participates in hydrogen bonding, an unfavorable interaction was also observed at this site. This suggests possible steric hindrance or electronic repulsion that may negatively impact the overall binding stability (Figure 2A). The 2D structure showed van der Waals interactions with several elastase residues. The active binding sites of L-phenylalanine on the elastase protein include Ser195, Ser214, Cys191, and Gln192, forming five hydrogen bonds and one unfavorable interaction (Figure 2B). Valine formed interactions with elastase at residues Ser195, Cys191, Gln192, Phe215, and Val216 (Figure 2C). n-Amylbenzene interacted with elastase at the Val216 residue, involving the C4 atom and the aromatic moiety (Figure 2D). 5-Phenylvaleric acid interacted with the active site residues Cys191, Ser195, Val216, and His57 (Figure 2E). 2-(3-Phenylpropyl) phenol compound bonded to elastase on residues CYS191, SER195, HIS57, VAL216, THR213, and ASP194 (Figure 2F).

Traumatin formed interactions with elastase at the active site residues Val216, Ser217, and Gln192 (Figure 2G). Retinoic acid, used as a reference inhibitor for elastase, demonstrated interactions with several amino acid residues of the enzyme. The active site residues involved in binding included Arg217, Val216, Val99, His57, Trp172, Phe215, and Ser195 (Figure 2H). The hydrophobicity profile revealed a high hydrophobicity value, while the hydrogen bonding analysis indicated the presence of a hydrogen bond acceptor.

**Figure 3:**
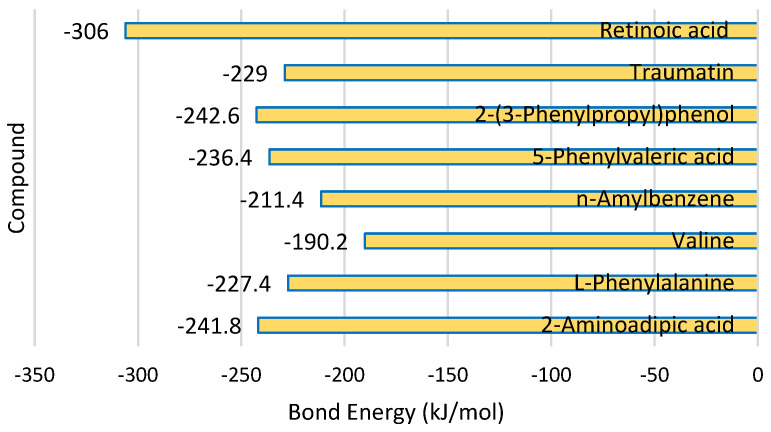
Bond energy between compound and protein elastase.

Based on the binding energy of the compounds with the elastase protein, retinoic acid showed the lowest energy, followed by the compound 2-(3-phenylpropyl)phenol (Figure 3). A lower energy bond will make for a stronger interaction between the compound and the elastase protein.

**Figure 4:**
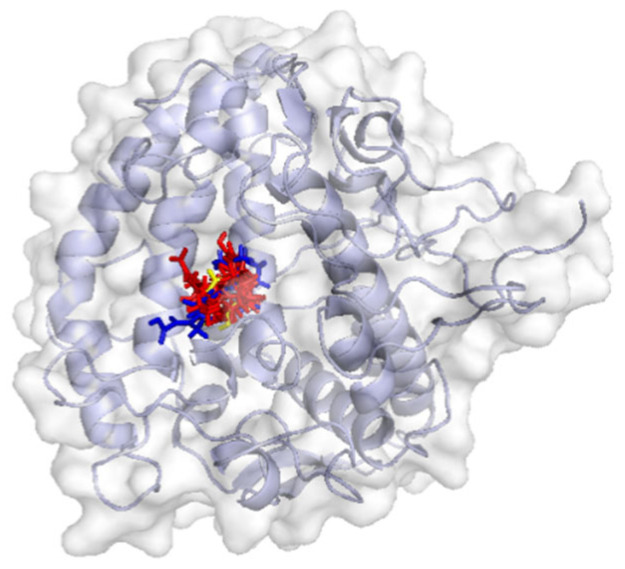
Target compound and tyrosinase interaction: gray represents tyrosinase protein, red represents the target compound, blue represents the inhibitor (native ligand), and yellow represents retinoic acid.

All target compounds demonstrated binding interactions with tyrosinase at the inhibitor region (Figure 4, Table 9). Notably, the Met280 residue was involved in the binding of 2-aminoadipic acid and valine, suggesting its role in selective ligand recognition. Val283 and His263 were consistently identified as interacting residues across all compounds except 2-aminoadipic acid, indicating a shared binding motif among the majority of the ligands. Additionally, Ala286 was observed at the active site of n-amylbenzene, 5-phenylvaleric acid, 2-(3-phenylpropyl)phenol, and traumatin, further supporting their potential as tyrosinase inhibitors through conserved molecular interactions.

**Figure 5:**
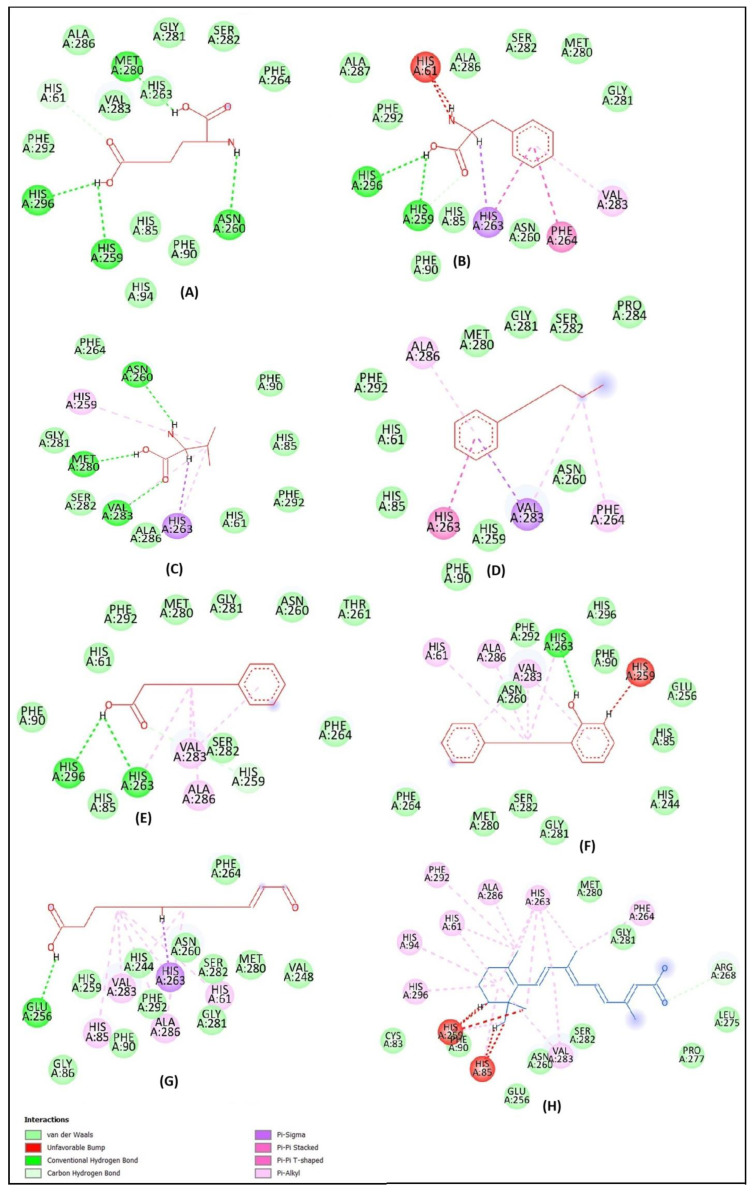
Two-dimensional diagram of the interaction between 2-aminoadipic acid (**A**), L-phenylalanine (**B**), valine (**C**), n-amylbenzene (**D**), 5-phenylvaleric acid (**E**), 2-(3-phenylpropyl)phenol (**F**), traumatin (**G**), and retinoic acid (**H**) with tyrosinase.

The 2-aminoadipic acid compound binds with tyrosinase through five hydrogen bonds at residues ASN260, MET280, HIS259, HIS296, and HIS61 (Figure 5A). L-Phenylalanine forms three hydrogen bonds, four hydrophobic interactions, two unfavorable bonds, and several van der Waals forces. The residues involved in the binding include HIS259, HIS296, HIS263, PHE264, VAL283, and HIS61 (Figure 5B). Valine interacts with tyrosinase through three hydrogen bonds and four hydrophobic interactions. The identified active site residues involved include VAL283, ASN260, MET280, HIS263, and HIS259 (Figure 5C). The compound n-amylbenzene interacts with tyrosinase through hydrophobic interactions at residues VAL283, HIS263, PHE264, and ALA286 (Figure 5D). The compound 5-phenylvaleric acid binds with tyrosinase at residues HIS263, HIS296, HIS259, VAL283, and ALA286 (Figure 5E). Meanwhile, the compound 2-(3-phenylpropyl)phenol forms a complex with the tyrosinase protein at the active site residues HIS263, VAL283, ALA286, HIS61, and HIS259 (Figure 5F). Traumatin inhibits the tyrosinase enzyme on amino acid residues such as GLU256, HIS263, VAL283, ALA286, HIS61, and HIS85 (Figure 5G). Retinoic acid, as a control, showed interaction with some residues, like ARG268, VAL283, ALA286, HIS61, HIS85, HIS94, HIS259, PHE264, PHE292, HIS296, and HIS263 (Figure 5H).

**Figure 6:**
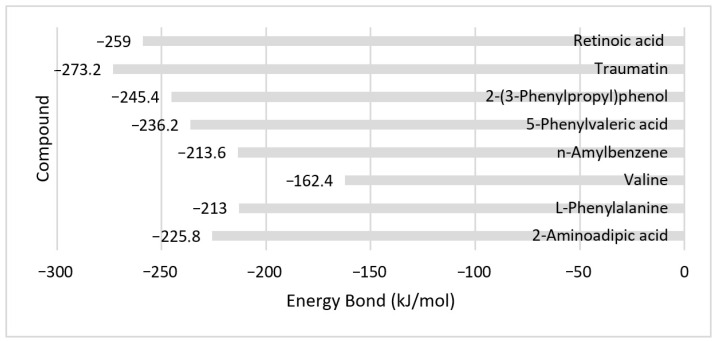
Bond energy of compound with tyrosinase protein.

Traumatin has the lowest bond energy with tyrosinase protein, followed by retinoic acid (Figure 6). A lower energy bond will make for a stronger interaction between the compound and the tyrosinase protein. Tyrosinase inhibitors work by binding to the enzyme and preventing it from performing its catalytic function. A lower (more negative) binding energy signifies a more stable and favorable interaction between the inhibitor molecule and the enzyme (tyrosinase).

## 3. Discussion

The extraction results showed that the fruit extract of *Eugenia uniflora* had the highest yield, although not significantly different from the seed extract, while the stem bark extract yielded the lowest. These differences in outcomes were potentially influenced by the varying chemical compositions characteristic of each specific plant part. Plant parts of interest, such as fruit, seed, or stem bark, are treated with an appropriate solvent to extract the phytochemicals. The extraction conditions can have different effects on the extraction yield of different plant parts. In another study of other species, extraction using ethanol solvent obtained similar results where the yield of stem bark extract (7.2%) was the lowest.

The qualitative phytochemical screening of bark, fruit, and seed extracts of *Eugenia uniflora* in this research showed the presence of terpenoids, alkaloids, flavonoids, saponins, and tannins. Several previous studies have demonstrated that *Eugenia uniflora* contains phytochemicals such as anthraquinones, steroids, triterpenes, flavonoids, saponin heterosides, and tannins. This is in line with other studies that screened *Eugenia uniflora* leaf extracts [30]. The fruits present various phytochemicals such as catechins, flavonols, proanthocyanidins, and carotenoids [31].

Phenolics and flavonoids are crucial phytochemicals responsible for antioxidant activity. The hydroxyl groups present in phenolic compounds are particularly effective at facilitating free radical scavenging. The antioxidant potency of flavonoids, in particular, depends on the number and position of their hydroxyl groups [32]. Several studies have indicated a strong correlation between high total phenolic content (TPC) and total flavonoid content (TFC) and anti-elastase and antioxidant activities [33,34,35]. Phenolic compounds are widely recognized for their antioxidant capacity. Their molecular structures, characterized by aromatic rings bearing one or more hydroxyl (-OH) groups, are fundamental to their efficacy. These hydroxyl groups are key to their ability to scavenge free radicals. The mechanism primarily involves the donation of a hydrogen atom or an electron to highly reactive free radicals (such as hydroxyl radicals, superoxide anions, and peroxyl radicals), thereby stabilizing the radical and breaking the chain reaction of oxidative stress. This process leads to the formation of a more stable, less reactive phenoxyl radical, which is often resonance-stabilized across the phenolic ring system, further enhancing its stability [36].

The antioxidant potency of flavonoids is highly dependent on specific structural features, including the number and position of hydroxyl groups, the presence of a C2-C3 double bond in conjugation with a 4-oxo group in the C-ring, and the catechol (ortho-dihydroxyl) structure in the B-ring. For instance, a higher number of hydroxyl groups generally enhances radical scavenging activity due to increased sites for hydrogen donation. The presence of hydroxyl groups at positions 3′ and 4′ (catechol moiety) in the B-ring, and at position 3 in the C-ring significantly contributes to their electron-donating ability and subsequent antioxidant power. This is because these specific arrangements allow for the formation of stable semiquinone radicals, crucial intermediates in the radical scavenging process [32].

Our research found that the seed extract exhibited the highest amounts of phenolics and flavonoids compared to the fruit and bark extracts. This aligns with previous research that proved that the seed extract of *E. involucrata* has a greater amount of phenolic compounds and antioxidant activity [35]. Seeds typically exhibit higher concentrations of flavonoids and polyphenols compared to other plant parts. This phenomenon is largely due to their integral role in protecting the developing embryo and providing essential nutritional reserves. Additionally, seeds serve as important dietary sources of minerals, accumulating these compounds during plant growth to support subsequent developmental needs. Therefore, *Eugenia uniflora* seed extracts have more flavonoid and phenolic compounds than fruit and bark extracts [37].

In this study, the dewandaru seed extract demonstrated strong anti-elastase activity with IC_50_ values of 114 µg/mL. A lower IC_50_ value indicates greater inhibitory activity [18,31]. Polyphenols can bind and inhibit digestive proteins. Kinetic measurements of porcine pancreatic elastase (PPE) activity were determined using Suc-(Ala)3-p-nitroanilide as a substrate and polyphenolic compounds as inhibitors. They can bind to enzymes through hydrogen interactions between the hydroxyl groups and their amino acid side chains to break down and inhibit the catalytic activity of enzymes [38]. High anti-elastase activity has been documented due to the presence of phenolic compounds (such as catechins, epicatechins, resveratrol, and procyanidin B2) and flavonoids (such as quercetin, kaempferol, and myricetin) in the extract or as single bioactive molecules. Furthermore, the presence of phenolic substances (carotenoids, flavonoids, and polyphenols) is often responsible for plants’ powerful antioxidant action [39]. A phytochemical analysis of the *E. uniflora* seed extract revealed the presence of key compounds, including ellagic acid, quercetin, and kaempferol [40].

Several compounds of *E. uniflora* seed extract that are associated with elastase inhibition activity are gallic acid, myricitrin, and myricetin. Gallic acid is known for its ability to suppress inflammation and oxidative stress [41]. Polyphenols like quercetin, myricitrin, and myricetin are able to form hydrogen bonds and bind to amino acid groups of the elastase. These will eliminate the catalytic activity site and cause the denaturation of the elastase enzyme. The binding process provokes a hydrophobic effect that produces complexes that are not soluble in water; then, elastase will be denatured [42]. This research shows that the seed extract may prevent premature aging of the skin induced by UV with the ability to increase the elasticity of the skin.

The elastase inhibitory activities of 2-(3-phenylpropyl)phenol and 5-phenylvaleric acid observed in this study show promising alignment with prior findings on effective elastase inhibitors. Both compounds demonstrated strong binding affinity toward key catalytic residues—HIS57, SER195, and VAL216—which are essential for the proteolytic activity of elastase [43]. Their molecular frameworks, which include hydrophobic alkyl chains and aromatic rings, contribute significantly to interaction stability through Pi–alkyl and hydrophobic contacts, in line with earlier structure–activity relationship (SAR) studies on elastase inhibitors [44].

In particular, 2-(3-phenylpropyl)phenol showed interaction with HIS57 and SER195, replicating binding profiles reported for known inhibitors such as retinoic acid and N-benzoyl-L-arginine ethyl ester (BAEE), where the aromatic moiety enhances Pi-stacking with the histidine imidazole ring [43]. The phenolic group in this compound also provides additional hydrogen bonding potential, which has been suggested to improve binding specificity [45].

Similarly, 5-phenylvaleric acid, with its extended aliphatic chain and terminal carboxyl group, mimics features of fatty-acid-derived inhibitors, which often leverage the flexibility of their side chains to achieve optimal alignment within the S1 pocket of elastase [46]. Its interactions with HIS57 and SER195 reinforce its potential to function as a competitive inhibitor, particularly when compared to naturally occurring elastase inhibitors like sivelestat or dipeptidyl boronic acids, which rely on covalent and non-covalent interactions at the same catalytic triad [47].

While both compounds displayed interaction energies slightly higher than retinoic acid, the differences were minimal, suggesting a potential for optimization. Importantly, their favorable pharmacokinetic predictions, including blood–brain barrier permeability and minimal cytochrome P450 inhibition, offer additional advantages for therapeutic development over some previously reported inhibitors with poor bioavailability or off-target effects [48].

2-(3-Phenylpropyl)phenol and 5-phenylvaleric acid present structural and pharmacological profiles that are consistent with effective anti-elastase scaffolds reported in prior literature. Their interactions with catalytically important residues and favorable docking scores underscore their potential as leads for further anti-inflammatory and anti-aging drug development.

Tyrosinase is a widely recognized strategy for managing hyperpigmentation conditions like melasma, freckles, and age spots. Tyrosinase, the key enzyme involved in melanogenesis, plays a central role in the synthesis of melanin pigments in the skin. One approach to anti-aging therapy involves the use of tyrosinase inhibitors—compounds that reduce the enzymatic activity of tyrosinase in melanin production [49]. In this study, in silico molecular docking revealed that all selected target compounds could bind to the active or allosteric inhibitor region of tyrosinase, suggesting a promising inhibitory effect. The involvement of critical amino acid residues such as His263, Val283, Ala286, and Met280 across multiple compounds is consistent with previous reports indicating that these residues as essential for substrate recognition and catalytic activity in the tyrosinase active site [50,51].

Among the tested ligands, 2-aminoadipic acid demonstrated the highest number of hydrogen bonds, highlighting its potential as a strong tyrosinase inhibitor. Hydrogen bonding is a critical determinant of ligand stability and specificity within the enzyme pocket [52]. Additionally, hydrophobic interactions such as π–π stacking and alkyl bonding further stabilized the ligand–enzyme complexes, particularly in compounds like traumatin and n-amylbenzene, which showed multiple non-polar interactions with the active site residues.

The docking results for retinoic acid, a known tyrosinase inhibitor, served as a benchmark. Its interaction profile—with the involvement of His263, Ala286, and Val283—was closely mimicked by several test compounds, particularly 5-phenylvaleric acid and 2-(3-phenylpropyl)phenol, suggesting a similar mechanism of action. These findings align with recent research on the structure–activity relationship (SAR) of flavonoid and phenolic inhibitors of tyrosinase [53]. Collectively, these results reinforce the hypothesis that naturally occurring compounds with specific binding affinities to key enzyme residues, like those found in *E. uniflora* seeds, can act as effective inhibitors. This opens exciting opportunities for developing alternative agents from safer, potentially less cytotoxic natural sources. However, to fully confirm their efficacy and safety for pharmaceutical or cosmeceutical applications, further validation through in vitro enzymatic inhibition assays and cytotoxicity evaluations is essential.

Our findings strongly indicate that *Eugenia uniflora* seed extract strongly showed the potential for both significant antioxidant, anti-elastase and anti-tyrosinase properties. These findings support the traditional uses of *Eugenia uniflora* and highlight its potential as a source of natural compounds for various health-promoting applications, particularly in areas related to oxidative stress management and skin health. However, further validation through in vitro enzymatic inhibition assays and cytotoxicity evaluations will be necessary to confirm the efficacy and safety of these compounds for pharmaceutical or cosmeceutical applications.

## 4. Materials and Methods

### 4.1. Plant Samples and Chemicals

*Eugenia uniflora* samples were collected in Depok, West Java. DPPH (2,2-diphenyl-1-picryl-hydrazyl) (Tokyo Chemistry Industry, Tokyo, Japan). The study also involved the use of porcine pancreatic elastase enzyme (≥99%), SANA substrate (N-succinyl-Ala-Ala-Ala-p-nitroanilide) (≥98%) (Sigma Aldrich, Darmstadt, Germany), Trizma base (HiMedia, Mumbai, India), TPTZ (2,4,6-tris(2-pyridyl)-triazine) (Sigma Aldrich, Darmstadt, Germany), and Folin–Ciocalteu (Sigma Aldrich, Steinheim, Germany); aluminum chloride (Merck, Darmstadt, Germany), iron (III) chloride hexahydrate, iron (II) sulfate heptahydrate, potassium dihydrogen phosphate, sodium acetate (Merck, Darmstadt, Germany), sodium hydroxide (Merck, Darmstadt, Germany), gallic acid (≥96%) (Merck, Darmstadt, Germany), quercetin (≥95%) (Sigma Aldrich, Darmstadt, Germany), ethanol pro analyze (Merck, Darmstadt, Germany), and methanol pro analyze (Merck, Darmstadt, Germany) and water pro injection (Pharmaceutical Laboratories, Bekasi, Indonesia).

### 4.2. Sample Preparation and Ultrasound-Assisted Extraction (UAE)

The bark and ripe fruits of *Eugenia uniflora* were gathered from Depok City. The fruits were cut in cross-section, and the seeds were removed from the fruit pericarp. The seeds were collected by removing the pericarp from red fruits. The seeds were left to dry naturally for seven days in the air. The fruit skin and pulp were freeze-dried and stored in the freezer.

Ten grams of bark, red ripe fruit, seed simplicia powder, and 70% hydroethanolic solvent were prepared in a sample–solvent ratio of 1:10. Both simplicia were extracted using the ultrasonic-assisted extraction (UAE) method with a Sonicator—Q2000 (QSonica, Newtown, NSW, USA) with a direct probe type immersed in 70% ethanol solvent medium at room temperature, a frequency of 20 kHz, and an extraction time of 30 min. The extracted samples were filtered and evaporated with a rotary evaporator to obtain fruit, seed, and bark extracts. Furthermore, the yield was calculated by comparing the extract’s weight with the initial simplicia’s weight.

### 4.3. Phytochemical Screening

Phytochemical screening was performed on the bark, fruit, and seed extracts following the protocols established in prior research [54]. The secondary metabolite compounds were identified using the reaction of color changing and precipitating for terpenoids, flavonoids, alkaloids, saponins, and tannins.

### 4.4. TPC Assay

The total phenolic content of the extracts was determined using Folin–Ciocalteu reagent. A mixture of 1 mL of each extract and 5 mL of 7.5% Folin–Ciocalteu diluted solution was prepared, shaken for 1 min, and left to stand for 8 min. Four milliliters of 1% sodium hydroxide solution was added, and then the mixture was incubated at room temperature for an hour in the dark. A UV-Vis spectrophotometer set at 730 nm was used to ascertain the absorbance value. The calibration plot was computed using gallic acid as the standard. A standard curve was established by employing a series of concentrations of gallic acid. The equation y = 0.0071x − 0.0145 was utilized to characterize the resulting curve, which had an R-squared value of 0.9971. The total phenolic content of each extract was determined in triplicate using the gallic acid calibration curve equation. The total phenol content of the extracts was quantified as milligrams of gallic acid equivalent per gram of the extract (mg GAE/g).

### 4.5. TFC Assay

A reaction was conducted with a volume of 0.5 mL of each extract solution in combination with 1.5 mL ethanol P, 0.1 mL 10% aluminum chloride, and 0.1 mL 1 M sodium carbonate. The yellow color in the mixture shows the possible presence of flavonoid molecules. After being vortexed, the mixtures were placed in an incubator at dark ambient temperature for a half hour. The absorption measurement was performed utilizing a UV-Vis spectrophotometer (T80+) set to a wavelength of 437 nm. The experiment was repeated 2 more times. A standard curve was generated using standard quercetin in serial concentrations. The equation representing the curve’s outcome was y = 0.0082x − 0.0402, with an R^2^ value of 0.999. Quercetin in a 5–100 mg/L concentration range was used as the standard to generate a calibration curve. The total flavonoid content was determined by using the standard curve equation, and the resulting values were expressed in milligrams of QE per gram of extract [55].

### 4.6. DPPH Antioxidant Assay

A certain volume of each extract in different concentration series solutions was added to the methanolic DPPH solution (0.3 mM). The methanol solvent was added to the mixture to make a total volume of 5 mL. After that, the samples were left out in the dark at 25 °C for 30 min for incubation. Next, a UV-Vis spectrophotometer was used to measure the sample’s absorbance at 517 nm. The procedure was carried out three times. The determination of the extract’s antioxidant bioactivity is stated as IC_50_. The IC_50_ value is the amount of the sample that can prevent as much as 50% DPPH radical activity. The inhibition percentage is obtained based on the DPPH radical scavenging capabilities of the sample solution in every concentration. The equation y = a + bx is determined by making a linear regression curve with x representing the sample concentration in g/mL units and y representing the inhibition percentage.

### 4.7. FRAP Assay

Iron (II) sulfate heptahydrate solution was used as a standard series solution to obtain a linear regression equation y = bx + a and produce the calibration curve. The concentration was calculated using the equation below.

$$C=\frac{\Delta A596\mathrm{nm}-a}{b}$$

ΔA is the difference between blank absorbance and sample absorbance. The solution was mixed with FRAP reagent and methanol, resulting in a final volume of 5 mL. The same procedure was carried out for the standard (ascorbic acid). The mixture solution was placed in a closed dark compartment for half an hour. A UV-Vis spectrophotometer was used to measure the absorbance of the sample at a wavelength of 596 nm. The percentage of Fe^3+^ to Fe^2+^ reduction by the sample in μmol FeSO_4_/g of dried extract is stated as the value of FRAP [56]. C is the sample concentration (μM), V is the sample volume (mL), Fp is the dilution factor, and m is the mass of sample (mg). The FRAP value was determined using the following equation.

$$FRAP\;value=\frac{C\times V\times Fp}{m}$$

### 4.8. Anti-Elastase Assay

Using color-changing observation, the UAE extracts of *E. uniflora* were evaluated for their antiaging properties. The elastase inhibition is determined by the appearance of a yellow color as a reaction between elastase and N-succinyl-Ala-Ala-Ala-p-nitroanilide (SANA). On a 96-well plate containing 125 µL of Tris–HCl buffer solution with an acidity level of 8.0, 30 μL of extracts (10–1000 μg/mL) were added, respectively, and 15 μL of porcine pancreas elastase (0.22 units/mL). The mixture was kept in an incubator (Memmert IN55) set at 25 °C for 20 min. Thirty microliters of SANA substrate solution with a concentration of 1.3 mM SANA was added to the previous mixture. The total volume of the 200 µL mixture was incubated again at the same temperature for 50 min. The absorption was measured at 405 nm using a microplate reader (GloMax^®^ Discover). The inhibitory activity of elastase by the extracts was quantified as the IC_50_ value. Quercetin was a positive control, and the extracts were tested 3 times [55]. The following inhibition percentage formula was used to determine the anti-elastase activity of each concentration:

$$\%Inhibition=\frac{A_{0}{-A}_{1}}{A_{0}}\times 100\%$$

A_0_ represents the blank absorbance, which is deducted from the blank control absorbance, while A1 represents the sample absorbance, which is deducted from the sample control absorbance. The inhibition percentage from the concentration series of sample solutions and positive controls will provide a linear regression curve of anti-elastase enzyme activity. The sample concentration is represented by x, and the inhibition percentage is represented by the y-axis to obtain the equation y = a + bx. The formula of the IC_50_ value calculation is presented below.

$${\mathrm{IC}}_{50}=\frac{50-a}{b}$$

### 4.9. LC-MS Study

The samples were centrifuged at 1400× *g* for 5 min to separate the supernatant from the pellet. The supernatant was collected and filtered using a PTFE filter with a size of 0.22 µm. The supernatant was injected for LC-HRMS analysis. MS grade MeOH was used as a blank sample solution for analysis. A liquid chromatography system (Thermo Scientific™ Vanquish™ UHPLC Binary Pump) (Thermo Scientific, Bremen, Germany) and Orbitrap (Thermo Scientific™ Q Exactive™ Hybrid Quadrupole-Orbitrap™) (Thermo Scientific, Bremen, Germany) high-resolution mass spectrometry were used in the analysis. An analytical column (Thermo Scientific’s Accucore™ Phenyl-Hexyl analytical column) (Thermo Scientific, Bremen, Germany) with a size of 100 mm × 2.1 mm ID × 2.6 µm was used for liquid chromatography. The mobile phases utilized were aquadest (MS-grade) containing 0.1% formic acid (A) and MS-grade methanol containing 0.1% formic acid (B), using a gradient method with a 0.3 mL/min flow rate. First, the mobile phase B was set to 5% and steadily rose to 90% in 16 min. Then, it remained at 90% for 4 min before returning to the previous state (5% B) for another 25 min. The temperature of the column was set at 40 °C, and the injection volume was 3 µL. The untargeted screening was carried out in complete MS/dd-MS2 acquisition mode, with either positive or negative ionization polarities/states. Nitrogen was utilized for sheath, auxiliary, and sweep gases, with arbitrary units of 32, 8, and 4, respectively. The voltage of the spray was 3.30 kV, the temperature of the capillary was 320 °C, and the auxiliary gas heater temperature had been adjusted to 30 °C. The scanning is held out in the range of 66.7–1000 *m/z*, with a resolution of 70,000 for full MS and 17,500 for dd-MS2 in both positive and negative ionization modes. The system was managed using XCalibur 4.4 software (Thermo Scientific, Bremen, Germany). The instrument was tuned and calibrated weekly in both ESI-positive and -negative modes using Thermo Scientific Pierce ESI ion calibration solutions (Waltham, MA, USA) to ensure optimal and robust instrumental performances throughout the analysis, including mass accuracy (<5 ppm), ion transfer, ion isolation, and instrumental sensitivity [56].

### 4.10. Preparation of Protein and Ligand Structures

The phytochemical compounds of the LC-MS list were screened according to the structure deposited in the database. Each of the compounds was predicted for its antiaging bioactivity using PASS Online (https://www.way2drug.com/passonline/, accessed on 31 January 2025). The analysis result was screened with the keywords of aging, tyrosinase, and elastase, and then the result of the screening was visualized using a heatmap (http://heatmapper.ca/expression/ (accessed on 31 January 2025)).

### 4.11. Target Protein Structure

The target protein structures of anti-elastase and anti-tyrosinase were obtained from Protein Data Bank (https://www.rcsb.org/ (accessed on 31st January 2025)). The protein structure of porcine pancreatic elastase (PDB ID 2H1U) was obtained from https://www.rcsb.org/structure/2H1U (accessed on 31st January 2025) [57], while the tyrosinase protein structure (PDB ID 2Y9X) was obtained from https://www.rcsb.org/structure/2Y9X (accessed on 31 January 2025) [58].

### 4.12. Prediction of Pharmacokinetics and Toxicity of Target Compound

The target compound structure (Table 6) was characterized according to pharmacokinetics (absorption, distribution, metabolism, excretion, and toxicity). Prediction was performed using the Deep-PK program (https://biosig.lab.uq.edu.au/deeppk/ (accessed on 31 January 2025)) [59].

### 4.13. Molecular Docking Simulation

Targeted compound structures (Table 6) interacted with protein targets elastase and tyrosinase. Docking simulation was held using Molegro Virtual Docker 5.0 [60]. The docking parameter used in Molegro virtual docker was the score function. MolDock Score [Grid]; grid resolution 0.30; algorithm MolDock SE; number of runs, 10, Max iteration, 1500; max population size, 50; pose generation energy threshold, 100; tries, 10–30; simplex evolution max steps, 300; neighbor distance factor, 1.00; multiple pose number of poses, 5; energy threshold, 0.00; cluster similar poses RMSD threshold, 1. The grids for tyrosinase docking were X = −9.94, Y = −28.34, Z = −44.44, radius 9, and cavity volume 49,152. The grids for elastase docking were X = −10.20; Y = 23.78; Z = 34.34; Radius 7.

The result of the docking with Molegro Virtual Docker version 5 was combined with protein (superimposed) using PyMol 2.2. The visualization docking shows the appearance of 3D and 2D and their interaction using Discovery Studio 21.1.1.

## 5. Conclusions

The ripe fruit UAE extract has the greatest antioxidant activity, which is supported by the highest content of phenolic and flavonoid compared to other UAE extracts. The ripe fruit has strong elastase inhibition activity, and it may be considered a potential natural ingredient for the development of new antiaging pharmaceutical products. The molecular docking analysis highlights several candidate compounds with dual inhibitory potential against both tyrosinase and elastase enzymes. Notably, 2-aminoadipic acid, L-phenylalanine, valine, and traumatin exhibited strong binding interactions with key active site residues of tyrosinase, suggesting their potential as effective agents for managing hyperpigmentation and other melanin-related conditions. In parallel, 2-(3-phenylpropyl)phenol and 5-phenylvaleric acid demonstrated robust interactions with catalytic residues of elastase, supporting their potential as anti-inflammatory and anti-aging agents. These findings offer valuable insight into the development of novel cosmeceutical or pharmaceutical formulations derived from natural or semi-synthetic sources and warrant further investigation through in vitro and in vivo validation studies.

## Figures and Tables

**Table 1:** Phytochemical screening of Dewandaru bark, fruit, and seed extract.

Sample	B	F	S
Terpenoids	++	+	−
Alkaloids	−	+	+
Tannins	+	+	+
Flavonoids	+	+	+
Saponins	−	+	++

B = bark, F = fruit, S = seed, ++ means = abundant, + = present, − = absent.

**Table 2:** Determination of TPC and TFC of *Eugenia uniflora* UAE extract.

Sample	TPC (mg GAE/g Extract) *	TFC (mg QE/g Extract) *
Fruit Extract	4.35 ± 0.39	0.65 ± 0.01
Seed Extract	11.55 ± 2.44	2.72 ± 0.04
Bark Extract	3.86 ± 0.10	0.99 ± 0.02

*n* = 3, TPC: total phenolic content, TFC: total flavonoid content, GAE: gallic acid equivalent, QE: quercetin equivalent, * dry weight.

**Table 3:** Antioxidant activities of *Eugenia uniflora* fruit, seed, bark extract, and control.

Sample	DPPH	FRAP
Fruit Extract	83.19 ± 0.57	210.99 ± 8.13
Seed Extract	5.71 ± 0.58	5181.67 ± 7.83
Bark Extract	10.75 ± 0.31	1101.62 ± 39.89
Quercetin	3.39 ± 0.09	1202.57 ± 44.73

*n* = 3.

**Table 4:** Anti-elastase activities of *Eugenia uniflora* seed extract.

C (µg/mL)	Abs	Blank	% inh	Mean ± SD	IC_50_ (µg/mL)	Regression Equation
23.44	0.522	0.801	36.39	38.34 ± 1.75		y = 0.087x + 40.03 R^2^ = 0.95
	0.516	0.801	39.81			
	0.521	0.801	38.81			
46.88	0.526	0.801	43.90	45.50 ± 1.71		
	0.481	0.801	47.30			
	0.518	0.801	45.30			
93.75	0.415	0.801	49.89	49.13 ± 0.76		
	0.482	0.801	49.13		114.14	
	0.491	0.801	48.38			
187.5	0.395	0.801	61.37	59.65 ± 1.95		
	0.428	0.801	60.04			
	0.418	0.801	57.54			
375	0.331	0.801	74.65	70.98 ± 6.36		
	0.299	0.801	63.64			
	0.312	0.801	74.65			

**Table 5:** LC-MS of ripe seed UAE extract.

No	RT	[M+H]+ m/z	Ion Fragments *m/z*	Formula	Name
1	0.936	191.015	173.008 191.055 192.022	C_6_H_8_O_7_	Citric acid
2	0.826	162.075	116.070 163.079 164.091	C_6_H_11_NO_4_	2-Aminoadipic acid
3	7.795	265.140	266.146	C_15_H_20_O_4_	(±)-Abscisic acid
4	15.297	359.310	360.320	C_21_H_42_O_4_	1-Stearoylglycerol
			361.320		
5	0.813	191.060	191.020	C_7_H_12_O_6_	D-(-)-Quinic acid
			192.022		
			193.059		
6	1.214	169.013	168.005	C_7_H_6_O_5_	Gallic acid
			170.017		
			171.017		
7	17.302	391.283	541.537	C_24_H_38_O_4_	Bis(2-ethylhexyl) phthalate
			540.534		
8	14.666	282.278	283.282	C_18_H_35_NO	Oleamide
9	5.555	198.127	197.854	C_14_H_15_N	Dibenzylamine
			198.203		
10	14.055	377.266	273.310	C21H38O4	1-Linoleoyl glycerol
			360.362		
			356.287		
11	5.567	465.103	464.279	C_21_H_20_O_12_	Myricitrin
			466.106		
			467.082		
12	0.779	175.119	176.091	C_6_H_14_N_4_O_2_	L-(+)-Arginine
			178.107		
13	14.452	256.263	255.645	C16H33NO	Hexadecanamide
			255.232		
			257.247		
			258.269		
14	5.565	319.044	319.155	C_15_H_10_O_8_	Myricetin
			320.048		
			321.049		
			322.125		
14	12.873	295.227	296.229	C_18_H_30_O_3_	9-Oxo-10(E),12(E)-
			301.140		octadecadienoic acid
15	14.647	357.299	359.305	C_21_H_40_O_4_	Monoolein
			358.303		
16	8.455	249.148	250.151 247.133	C_15_H_20_O_3_	6-Hydroxy-5a,9-dimethyl-3-methylene-3a,4,5,5a,6,7,9a,9b-octahydronaphtho[1,2-b]furan-2(3H)-one
17	9.707	307.154	308.157 303.156	C_17_H_22_O_5_	9a-Hydroxy-3,8a-dimethyl- 5-methylene-2-oxo-2,4,4a,5,6,7,8,8a,9,9a-decahydronaphtho[2,3-b] furan-8-yl acetate
18	5.746	253.179	252.087	C_15_H_24_O_3_	NP-008095
			254.183		
			255.062		
19	10.827	291.159	293.165	C_17_H_22_O_4_	NP-000295
			314.144		
20	1.627	127.039	123.117	C_6_H_6_O_3_	5-Hydroxymethyl-2-
			124.039		furaldehyde
21	12.448	279.232	280.235	C_18_H_30_O_2_	α-Eleostearic acid
			282.279		
22	0.835	118.086	119.089 120.065	C_5_H_11_O_2_	Valine
23	7.670	179.106	180.109 181.014	C_11_H_14_O_2_	5-Phenylvaleric acid
24	1.337	166.086	167.089	C_9_H_11_O_2_	L-Phenylalanine
			168.065		
25	8.902	387.166	388.169 389.171	C_11_H_16_	n-Amylbenzene
26	8.900	195.137	196.141 209.153 213.148	C_12_H_20_O_3_	Traumatin
27	8.759	213.127	214.131 214.577	C_15_H_16_O	2-(3-Phenylpropyl)phenol

**Table 6:** Pharmacokinetic characteristics of target compounds.

PK and Toxicity	Property	Unit	5-PVA	V	L-PA	n-AB	2-AAA	T	2-(3-PP)P	RA
Absorption	Caco-2 (log Paap)	log Paap	−4.9	−5.61	−4.94	−4.2	−5.91	−4.64	−4.82	−4.48
	Human Oral Bioavailability 20%	Category (BA/Non-BA)	BA	BA	BA	BA	BA	Non	BA	BA
	Human Intestinal Absorption	Category (A/Non-A)	A	A	A	A	A	A	A	A
	Madin–Darby Canine Kidney	cm/s	−3.88	−1.68	−4.38	−3.76	−4.63	−4.03	−4.32	−4.53
	Human Oral Bioavailability 50%	Category (BA/Non-BA)	BA	N	N	BA	N	N	BA	N
	P-Glycoprotein Inhibitor	Category (Inhibitor/Non)	N	N	N	N	N	N	N	N
	P-Glycoprotein Substrate	Category (Substrate/Non)	N	N	N	N	N	N	N	N
	Skin Permeability	log Kp	−2.99	−3.15	−1.56	−3.45	−2.57	−2.12	−2.86	−2.86
Distribution	Blood–Brain Barrier (Central Nervous System)	log PS	−1.76	−3.8	−2.36	−2.4	−3.66	−2.06	−1.42	−2.32
	Blood–Brain Barrier	Category (Penetrating/Non)	P	P	P	P	P	P	P	P
	Fraction Unbound (Human)	free proportion	0.8	0.46	−0.37	1.18	−0.09	0.51	1.31	1.17
	Plasma Protein Binding	therapeutic index	46.9	1.58	52.98	26.62	4.57	32.95	77.92	57.55
	Steady-State Volume of Distribution	log VDss	0.68	0.59	0.3	4.22	0.46	0.94	2.47	2.02
Metabolism	Breast Cancer Resistance Protein	Category (Inhibitor/Non)	N	N	N	N	N	N	N	I
	CYP 1A2 Inhibitor	Category (Inhibitor/Non)	N	N	N	I	N	N	I	N
	CYP 1A2_substrate	Category (Substrate/Non)	N	N	N	S	N	N	S	N
	CYP 2C19 Inhibitor	Category (Inhibitor/Non)	N	N	N	I	N	N	I	N
	CYP2C19 substrate	cyp2c19_substrate	N	N	N	N	N	N	N	N
	CYP 2C9 Inhibitor	Category (Inhibitor/Non)	N	N	N	I	N	N	I	I
	CYP 2C9 Substrate	Category (Substrate/Non)	N	S	S	N	N	S	S	S
	CYP2D6 Inhibitor	Category (Inhibitor/Non)	N	N	N	N	N	N	N	I
	CYP2D6 Substrate	Category (Substrate/Non)	N	N	N	S	N	N	S	N
	CYP 3A4 Inhibitor	Category (I/Non)	N	N	N	N	N	N	N	N
	CYP 3A4 Substrate	Category (S/Non)	N	N	N	N	N	N	S	S
	OATP1B1	Category (Inhibitor/Non)	N	N	N	N	N	N	N	I
	OATP1B3	Category (Inhibitor/Non)	N	N	N	N	N	N	N	N
Excretion	Clearance	Log (ml/min/kg)	5.6	1.89	9.08	7.26	−1.01	−0.17	6.93	−2.16
	Organic Cation Transporter 2	Category (Inhibitor/Non)	N	N	N	N	N	N	N	N
	Half-Life of Drug	Category (Half-life ≥ 3 hs/Half-life < 3 hs)	<3hs	<3hs	<3hs	<3hs	≥3hs	≥3hs	<3hs	<3hs

5 PVA = 5-phenylvaleric acid, V = valine, L-PA = L-phenylalanine, n-AB = n-amylbenzene, 2 AAA = 2-aminoadipic acid, T = traumatin, 2-(3-PP) P = 2-(3-phenylpropyl)phenol, RA = retinoic acid, BA = bioavailable A = absorbed, P = penetrable, S = substrate, I = inhibitor, N = Non.

**Table 7:** Toxicity of target compounds.

Property	5-PVA	V	L-PA	n-AB	2-AAA	T	2-(3-PP)P	RA
AMES Mutagenesis	√	√	√	√	√	√	√	√
Avian	√	√	√	√	√	√	√	√
Bee	√	X	√	√	√	√	√	√
Bioconcentration Factor, log10 (L/kg)	0.27	−1.19	−0.43	2.68	−0.22	−0.05	1.85	0.87
Biodegradation	X	X	X	√	X	X	√	√
Carcinogenesis	√	√	√	X	√	√	√	√
Crustacean	√	√	√	X	√	X	X	X
Liver Injury I (DILI)	√	√	X	√	√	√	√	√
Eye Corrosion	√	√	√	X	X	X	√	√
Eye irritation	X	X	X	X	X	X	X	X
Maximum Tolerated Dose, log mg/kg/day	1.02	2.21	1.98	0.3	1.8	1.5	1.0	0.38
Liver Injury II	X	√	X	X	√	√	X	X
hERG Blockers	√	√	X	√	√	√	√	√
Daphnia Maga, −log10 [(mg/L)/(1000*MW)]	3.57	3.91	3.46	5.02	2.32	4.9	5.48	4.05
Micronucleus	√	√	X	√	√	√	√	√
NR-AhR	√	√	√	√	√	√	√	√
NR-AR	√	√	√	√	√	√	√	√
NR-AR-LBD	√	√	√	√	√	√	√	√
NR-Aromatase	√	√	√	√	√	√	√	√
NR-ER	√	√	√	√	√	√	√	√
NR-ER-LBD	√	√	√	√	√	√	√	X
NR-GR	√	√	√	√	√	√	√	√
NR-PPAR-gamma	√	√	√	√	√	√	√	X
NR-TR	√	√	√	√	√	√	√	X
T. Pyriformis, −log10[(mg/L)/(1000*MW)]	3.06	−0.25	1.25	4.37	3.13	4.52	5.06	6.12
Rat (Acute), log[1/(mol/kg)]	1.91	1.7	2.06	1.64	1.3	1.95	1.87	2.07
Rat (Chronic Oral), log(mg/kg_bw/day)	2.09	2.03	2.17	2.09	2.04	2.06	1.93	2.26
Fathead Minnow, −log10[(mg/L)/(1000*MW)]	3.94	3.4	3.62	3.97	3.48	3.94	4.43	5.18
Respiratory Disease	√	√	√	√	√	X	√	X
Skin Sensitization	X	X	√	X	X	X	X	X
SR-ARE	√	√	√	√	√	√	X	X
SR-ATAD5	√	√	√	√	√	√	√	√
SR-HSE	√	√	√	√	√	√	√	X
SR-MMP	√	√	√	√	√	√	X	X
SR-p53	√	√	√	√	√	√	√	√

√ = safe, X = toxic.

**Table 8:** Interactions of target compounds with elastase.

Compound	CID	Interaction	Distance (A)	Bond	Type of Bond
2-Aminoadipic acid	469	:10:H8-A:SER195:OG	2.10548	Hydrogen Bond	Conventional Hydrogen Bond
		:10:H10-A:SER195:OG	1.80197	Hydrogen Bond	Conventional Hydrogen Bond
		:10:H11-A:VAL216:O	1.68497	Hydrogen Bond	Conventional Hydrogen Bond
		A:GLN192:NE2-:10:O4	3.15798	Hydrogen Bond	Conventional Hydrogen Bond
		:10:H11-A:VAL216:N	2.69377	Unfavorable	Unfavorable Donor–Donor
L-Phenylalanine	6140	:10:H6-A:SER195:OG	2.57393	Hydrogen Bond	Conventional Hydrogen Bond
		:10:H6-A:SER214:O	2.63281	Hydrogen Bond	Conventional Hydrogen Bond
		:10:H11-A:CYS191:O	1.59077	Hydrogen Bond	Conventional Hydrogen Bond
		:10:H3-A:SER195:OG	2.58541	Hydrogen Bond	Carbon–Hydrogen Bond
		A: GLN192:NE2-:10	3.89193	Hydrogen Bond	Pi–Donor Hydrogen Bond
		:10:H11-A:CYS191:N	2.55659	Unfavorable	Unfavorable Donor–Donor
Valine	6287	:10:H9-A:SER195:OG	2.28235	Hydrogen Bond	Conventional Hydrogen Bond
		:10:H10-A:CYS191:O	2.42935	Hydrogen Bond	Conventional Hydrogen Bond
		:10:H11-A:SER195:OG	2.22809	Hydrogen Bond	Conventional Hydrogen Bond
		A:GLN192:NE2-:10:O2	3.25682	Hydrogen Bond	Conventional Hydrogen Bond
		:10:H2-A:SER195:OG	2.28054	Hydrogen Bond	Carbon–Hydrogen Bond
		A: PHE215:CA-:10:O1	3.47172	Hydrogen Bond	Carbon–Hydrogen Bond
		:10:C1-A:VAL216	4.02234	Hydrophobic	Alkyl
		:10:H10-A:SER195:N	2.56414	Unfavorable	Unfavorable Donor–Donor
n-Amylbenzene	10,864	:10:C4-A:VAL216	4.00007	Hydrophobic	Alkyl
		:10-A:VAL216	5.41977	Hydrophobic	Pi–Alkyl
5-Phenylvaleric acid	16,757	:10:H14-A:CYS191:O	1.68264	Hydrogen Bond	Conventional Hydrogen Bond
		A:SER195:N-:10:O1	2.78092	Hydrogen Bond	Conventional Hydrogen Bond
		A:SER195:OG-:10	3.56553	Hydrogen Bond	Pi–Donor Hydrogen Bond
		:10-A:VAL216	5.28161	Hydrophobic	Alkyl
		A: HIS57-:10	5.28158	Hydrophobic	Pi–Alkyl
2-(3-Phenylpropyl)phenol	572,468	:10:H16-A:CYS191:O	1.82042	Hydrogen Bond	Conventional Hydrogen Bond
		A:SER195:N-:10:O1	2.82822	Hydrogen Bond	Conventional Hydrogen Bond
		A:SER195:OG-:10:O1	2.79543	Hydrogen Bond	Conventional Hydrogen Bond
		A: HIS57-:10	4.91782	Hydrophobic	Pi–Pi Stacked
		:10-A:VAL216	4.92124	Hydrophobic	Alkyl
		:10-A:VAL216	4.3249	Hydrophobic	Pi–Alkyl
		:10:C14-A:THR213:CG2	2.06988	Unfavorable	Unfavorable Bump
		:10:H14-A:THR213:CG2	1.31125	Unfavorable	Unfavorable Bump
		:10:H16-A:ASP194:N	2.64189	Unfavorable	Unfavorable Donor–Donor
Traumatin	5,312,889	:10:H19-A:VAL216:O	2.23678	Hydrogen Bond	Conventional Hydrogen Bond
		:10:H19-A:SER217:O	2.33672	Hydrogen Bond	Conventional Hydrogen Bond
		A:GLN192:NE2-:10:O2	2.99847	Hydrogen Bond	Conventional Hydrogen Bond
		:10-A:VAL216	4.75325	Hydrophobic	Alkyl
		:10-A:VAL216	4.56525	Hydrophobic	Alkyl
Retinoic acid	444,795	A:ARG217A:CD-:10:O1	3.34058	Hydrogen Bond	Carbon–Hydrogen Bond
		:10:C7-A:VAL216	2.87356	Hydrophobic	Alkyl
		:10:C8-A:VAL216	4.45649	Hydrophobic	Alkyl
		:10-A:VAL216	5.03321	Hydrophobic	Alkyl
		:10:C13-A:ARG217A	3.79967	Hydrophobic	Alkyl
		:10:C18-A:VAL99	5.46514	Hydrophobic	Alkyl
		A: HIS57-:10:C10	4.98999	Hydrophobic	Pi–Alkyl
		A: TRP172-:10:C18	5.46878	Hydrophobic	Pi–Alkyl
		A: PHE215-:10:C18	4.6441	Hydrophobic	Pi–Alkyl
		:10:C3-A:SER195:OG	2.11805	Unfavorable	Unfavorable Bump
		:10:H3-A:SER195:OG	1.09989	Unfavorable	Unfavorable Bump
		:10:H8-A:VAL216:CG2	1.80531	Unfavorable	Unfavorable Bump

**Table 9:** Interactions between target compounds and tyrosinase protein.

Compound	CID	Interaction	Distance (A)	Bond	Bond Type
2-Aminoadipic acid	469	:10:H9-A:ASN260:O	2.59891	Hydrogen Bond	Conventional Hydrogen Bond
		:10:H10-A:MET280:O	2.2952	Hydrogen Bond	Conventional Hydrogen Bond
		:10:H11-A:HIS259:NE2	2.88339	Hydrogen Bond	Conventional Hydrogen Bond
		:10:H11-A:HIS296:NE2	2.16919	Hydrogen Bond	Conventional Hydrogen Bond
		A:HIS61:CE1-:10:O4	3.0639	Hydrogen Bond	Carbon–Hydrogen Bond
L-Phenylalanine	6140	:10:H11-A:HIS259:NE2	1.95423	Hydrogen Bond	Conventional Hydrogen Bond
		:10:H11-A:HIS296:NE2	227029	Hydrogen Bond	Conventional Hydrogen Bond
		A: HIS259:CE1-:10:O2	3.12422	Hydrogen Bond	Carbon–Hydrogen Bond
		:10:H3-A:HIS263	2.43753	Hydrophobic	Pi–Sigma
		A: HIS263-:10	4.09972	Hydrophobic	Pi–Pi Stacked
		A: PHE264-:10	4.88035	Hydrophobic	Pi–Pi T-shaped
		:10-A:VAL283	5.23036	Hydrophobic	Pi–Alkyl
		A:HIS61:CE1-:10:N1	222005	Unfavorable	Unfavorable Bump
		A:HIS61:CE1-:10:H7	1.49841	Unfavorable	Unfavorable Bump
Valine	6287	A:VAL283:N-:10:O2	3.0412	Hydrogen Bond	Conventional Hydrogen Bond
		:10:H10-A:ASN260:OD1	2.73625	Hydrogen Bond	Conventional Hydrogen Bond
		:10:H11-A:MET280:O	1.80915	Hydrogen Bond	Conventional Hydrogen Bond
		:10:H2-A:HIS263	2.50188	Hydrophobic	Pi–Sigma
		:10:C1-A:VAL283	3.81457	Hydrophobic	Alkyl
		A: HIS259-:10:C1	5.47901	Hydrophobic	Pi–Alkyl
		A: HIS263-:10:C1	4.38926	Hydrophobic	Pi–Alkyl
n-Amylbenzene	10,864	A:VAL283:CG2-:10	3.86529	Hydrophobic	Pi–Sigma
		A:HIS263-:10	3.67353	Hydrophobic	Pi–Pi Stacked
		:10:C4-A:VAL283	4.94484	Hydrophobic	Alkyl
		A:PHE264-:10:C4	5.20499	Hydrophobic	Pi–Alkyl
		:10-A:ALA286	4.5319	Hydrophobic	Pi–Alkyl
5-Phenylvaleric acid	16,757	:10:H14-A:HIS263:NE2	3.00302	Hydrogen Bond	Conventional Hydrogen Bond
		:10:H14-A:HIS296:NE2	2.54051	Hydrogen Bond	Conventional Hydrogen Bond
		A:HIS259:CE1-:10:O2	3.25609	Hydrogen Bond	Carbon–Hydrogen Bond
		A: VAL283-:10	4.15	Hydrophobic	Alkyl
		A: ALA286-:10	3.60867	Hydrophobic	Alkyl
		A: HIS263-:10	3.82739	Hydrophobic	Pi–Alkyl
		:10-A:VAL283	5.33656	Hydrophobic	Pi–Alkyl
2-(3-Phenylpropyl)phenol	572,468	:10:H16-A:HIS263:NE2	2.10946	Hydrogen Bond	Conventional Hydrogen Bond
		:10-:10	4.02915	Hydrophobic	Pi–Pi Stacked
		A: VAL283-:10	3.96369	Hydrophobic	Alkyl
		A: ALA286-:10	3.60936	Hydrophobic	Alkyl
		A: HIS61-:10	5.49858	Hydrophobic	Pi–Alkyl
		A: HIS263-:10	3.74104	Hydrophobic	Pi–Alkyl
		:10-A:VAL283	4.41892	Hydrophobic	Pi–Alkyl
		:10-A:VAL283	5.14575	Hydrophobic	Pi–Alkyl
		A:HIS259:CE1-:10:H10	1.77522	Unfavorable	Unfavorable Bump
Traumatin	5,312,889	:10:H19-A:GLU256:OE2	2.10595	Hydrogen Bond	Conventional Hydrogen Bond
		:10:H2-A:HIS263	2.30065	Hydrophobic	Pi–Sigma
		A: VAL283-:10	4.42447	Hydrophobic	Alkyl
		A:VAL283-:10	3.68636	Hydrophobic	Alkyl
		A:ALA286-:10	4.55147	Hydrophobic	Alkyl
		A:ALA286-:10	4.92503	Hydrophobic	Alkyl
		A:HIS61-:10	5.06211	Hydrophobic	Pi–Alkyl
		A:HIS85-:10	4.59661	Hydrophobic	Pi–Alkyl
		A:HIS263-:10	4.30442	Hydrophobic	Pi–Alkyl
Retinoic acid	444,795	A:ARG268:CD-:10:O2	3.63325	Hydrogen Bond	Carbon–Hydrogen Bond
		A:VAL283-:10	5.29642	Hydrophobic	Alkyl
		A:VAL283-:10	4.09657	Hydrophobic	Alkyl
		A:ALA286-:10:C10	2.90699	Hydrophobic	Alkyl
		A:HIS61-:10	4.84395	Hydrophobic	Pi–Alkyl
		A:HIS61-:10:C10	3.91738	Hydrophobic	Pi–Alkyl
		A:HIS85-:10	4.2203	Hydrophobic	Pi–Alkyl
		A:HIS94-:10	5.25314	Hydrophobic	Pi–Alkyl
		A:HIS259-:10:C8	4.98592	Hydrophobic	Pi–Alkyl
		A:HIS263-:10	4.34395	Hydrophobic	Pi–Alkyl
		A:HIS263-:10:C7	4.77534	Hydrophobic	Pi–Alkyl
		A:HIS263-:10	4.66221	Hydrophobic	Pi–Alkyl
		A:HIS263-:10:C10	3.75815	Hydrophobic	Pi–Alkyl
		A:HIS263-:10:C13	4.69426	Hydrophobic	Pi–Alkyl
		A:PHE264-:10:C13	3.79419	Hydrophobic	Pi–Alkyl
		A:PHE292-:10:C10	4.99287	Hydrophobic	Pi–Alkyl
		A:HIS296-:10	5.04505	Hydrophobic	Pi–Alkyl
		A:HIS85:CD2-:10:C8	2.31781	Unfavorable	Unfavorable Bump
		A:HIS85:CD2-:10:H12	1.42596	Unfavorable	Unfavorable Bump
		A:HIS259:CE1-:10:C2	2.03096	Unfavorable	Unfavorable Bump
		A:HIS259:CE1-:10:C7	2.30287	Unfavorable	Unfavorable Bump
		A:HIS259:CE1-:10:H2	1.38313	Unfavorable	Unfavorable Bump
Inhibitor (native ligand)		:10:H1-A:MET280:O	1.79416	Hydrogen Bond	Conventional Hydrogen Bond
		:10:H2-A:MET280:O	2.04212	Hydrogen Bond	Conventional Hydrogen Bond
		A:VAL283:CG2-:10	3.88696	Hydrophobic	Pi–Sigma
		A:HIS263-:10	3.65091	Hydrophobic	Pi–Pi Stacked
		:10-A:ALA286	4.95416	Hydrophobic	Pi–Alkyl

## Data Availability

Data are contained within the article.
